# Supplementary material for: Efficacy and safety of cisplatin + docetaxel + 5-FU + leucovorin + methotrexate and epirubicin combination chemotherapy for advanced esophageal cancer
Source: PLoS One. 2025 Jun 26;20(6):e0326056. doi: 10.1371/journal.pone.0326056 (PMC12200775; doi:10.1371/journal.pone.0326056)
Supplement: Attachment — Submitted filename: response to reviewer.docx [file pone.0326056.s002.docx]

Dear Editor and Reviewers

Thank you for spending your valuable time to review our paper. Fortunately, this paper could be submitted to PLOS one. We were grateful to Editors and Reviewers to review this paper and make some useful recommendation. That made this paper more valuable. It was our honor to have an opportunity to publish in PLOS one. We have considered the review’s comment and replied

them as below.

Review Comments to the Author

Reviewer #1: Dear Authors,
This is an interesting and novel research, and I appreciated the opportunity to review it. I commend your valuable effort to advance the scientific understanding of this significant health concern. Below, I have outlined my suggestions for enhancing this manuscript.

1. The ratio of case and control groups, as well as not-matched groups for confounding factors, result in a considerable selection bias of the study that highly affects the power of the study.

Thanks for your suggestion. Since the CDFLME regimen was applied to esophageal cancer for the first time, we conducted the study on a small scale and attempted to minimize differences between the two groups. However, the issue of a limited sample size and selection bias could not be fully addressed. In the future, we plan to include a larger number of patients to enhance the validity and statistical power of the study.

2. Although I believe that language should not be a barrier to knowledge transfer, I would ask the authors to improve the writing of the paper.

Thanks for your suggestion. We will make every effort to improve the clarity and quality of the writing in the manuscript.

3. Please include the number of controls in the abstract as well.

Thanks for your suggestion. We will include the number of controls in the abstract.

4. In a part of the introduction section, CDFLEM is inconsistent with this in other parts of the manuscript (CDFLME). Additionally, please define it when first used.

Thanks for your suggestion. We will correct this error and provide a definition when the term is first introduced in the manuscript.

5. “Pathology” is the correct term for examining tissue biopsies, including histological analysis. Please substitute it with histology.

Thanks for your suggestion. We will correct this error and make every effort to improve the quality of the writing in the manuscript.

6. Please outline the target organs for metastasis that you considered concerning progressive disease.

Thanks for your suggestion. When the tumor exhibits distant metastasis, including the spread of cancer cells to other organs such as the lungs, liver, bones, or brain, it is considered disease progression. We will provide additional clarification in the Methods section.

7. Please rearrange and separate the paragraphs in the introduction section based on the subject change.

Thanks for your suggestion, we will rearrange and separate the paragraphs in the introduction section based on the subject change.

8. In some parts of the manuscript, including keywords and the body of the text, “regime” is mentioned instead of regimen.

Thanks for your suggestion. We will correct this error and make every effort to improve the quality of the writing in the manuscript.

9. Please define SCCHN when first used. Furthermore, this is inconsistent with other manuscript parts (HNSCC).

Thanks for your suggestion. We will correct this error and standardize the terminology to HNSCC throughout the manuscript

10. Please provide a reference for categorizing response levels and other definitions in the methods section.

Thanks for your suggestion. We have cited RECIST 1.1 in the Methods section as the reference for evaluating response levels.

11. Please provide a reference for the treatment schedule.

Thanks for your suggestion. This treatment schedule was based on Dr Lin's weekly CDFLME induction chemotherapy for patients with locally advanced HNSCC. Given the favorable performance of cisplatin-based regimens in esophageal cancer patients, we adapted this regimen and applied it on a small scale to esophageal cancer patients. We will include this reference in the Discussion section and cite it as [17]

12. Please clarify whether informed consent was obtained, as the current statement is unclear.

In this retrospective study, the requirement for informed consent was waived by the Institutional Review Board (IRB) of Changhua Christian Hospital (IRB number: 231101). This waiver was granted because the study involved de-identified data collected from medical records and posed minimal risk to the participants. The approval for the study, including the waiver of informed consent, was obtained on November 21, 2023.

13. Please clarify that all monitoring visits were completed before the start of the study and that the data was obtained from the hospital.

Thanks for your suggestion. All monitoring visits were completed prior to the commencement of this retrospective study. The data used in this study were obtained from the medical records of Changhua Christian Hospital, ensuring accuracy and completeness.

14. The sentence “The results of the multivariate analysis confirmed that the CDFLME regimen was beneficial to overall survival” is an interpretation of the statistical analysis results, which is better stated in the discussion section.

Thank you for pointing this out. We will revise the manuscript by moving the sentence, "The results of the multivariate analysis confirmed that the CDFLME regimen was beneficial to overall survival," to the Discussion section, where it will be better aligned with the interpretation of the statistical analysis results.

15. “to our knowledge” at the beginning of the discussion section is inappropriate for the sentence.

Thank you for pointing this out. We will revise the sentence by removing "to our knowledge" and rephrasing it to ensure clarity and appropriateness in the context of the discussion section.

16. Please place figures in the results section.

Thank you for your suggestion. We will revise the manuscript by relocating the figures to the Results section to ensure proper alignment with the presentation of data.

Reviewer #2:

1. When authors stated (In current research, there are few studies that explore the efficacy and safety of this type of head and neck cancer chemotherapeutic regimens in advanced esophageal cancer patients). Can you please cite these studies?

Thank you for your comment. We acknowledge this limitation and will revise the manuscript to include relevant references that support our statement.

If there are no directly related studies available, we will clarify this point and provide context to highlight the gap in current research. We will cite these studies as references 16, 17, and 18.

2. Can you confirm all grammars and typos' issues?.

Thank you for your suggestion. We will thoroughly review the manuscript to ensure that all grammatical errors and typographical issues are corrected

Reviewer #3:

1. This is a retrospective study with a relatively small sample size (94 patients, with only 13 in the CDFLME group), which limits the statistical power and generalizability. A prospective, randomized controlled trial with a larger cohort would provide more robust evidence.

Thank you for your feedback. We acknowledge the limitation of the small sample size in this retrospective study, particularly the small number of patients in the CDFLME group. We have addressed this limitation in the manuscript. In the future, we plan to conduct a prospective, randomized controlled trial with a larger cohort to provide more robust and generalizable evidence.

2. The CDFLME group had a higher proportion of patients with better performance status (ECOG 0-2), which could have influenced the improved survival rates and outcomes. Addressing potential selection bias would improve the study's credibility.

Thank you for highlighting this point. We acknowledge that the higher proportion of patients with better performance status (ECOG 0-2) in the CDFLME group could have influenced the improved survival rates and outcomes. We have addressed this potential selection bias as a limitation in the manuscript and discussed its impact on the study's findings. In future studies, we aim to minimize selection bias by using randomized controlled designs to improve the credibility and reliability of the results.

3. While the study compares the novel CDFLME regimen to the standard of regimen, it doesn’t consider other potentially relevant combinations, such as those including immune checkpoint inhibitors, which have shown efficacy in advanced esophageal cancer.

Thank you for your valuable comment. In our study, both groups were treated according to the NCCN guidelines. For patients meeting the criteria for immunotherapy, immune checkpoint inhibitors were administered regardless of whether they received neoadjuvant chemoradiotherapy (NCRT). This approach ensures consistency in treatment decisions and avoids bias introduced by treatment selection. We will clarify this point in the manuscript.

4. The study focuses on survival and response rates but does not evaluate the patients' quality of life. Including metrics on quality of life would provide a more comprehensive view of the treatment’s impact.

Thank you for your insightful comment. We acknowledge that our study primarily focuses on survival and response rates and does not include quality-of-life metrics. This is a limitation of the current study, and we will address it in the manuscript. In future research, we plan to incorporate validated quality-of-life assessment tools to provide a more comprehensive evaluation of the treatment's impact on patients.

5. Although toxicity is briefly discussed, more in-depth analysis and detailed reporting (such as long-term toxicity and cumulative side effects) would be valuable to assess the regimen’s safety profile.

Thank you for your valuable suggestion. While our current study provides a brief discussion on toxicity, we acknowledge the importance of a more in-depth analysis and detailed reporting, including long-term toxicity and cumulative side effects, to comprehensively assess the safety profile of the regimen. We will revise the discussion section to address this limitation and include it as a recommendation for future research

6. A future study should ideally be a randomized controlled trial with a larger and more diverse patient population to validate these findings and minimize bias.

Thank you for your valuable suggestion. We completely agree that a future study should ideally be a randomized controlled trial with a larger and more diverse patient population to validate our findings and minimize bias. We have acknowledged this point as a limitation in our current study and plan to design future research accordingly.

7. Including other treatment regimens, especially those with newer therapeutic options, would provide a more comprehensive comparison and reflect current standards in oncology.

Thanks for your suggestion. We agree that those newer therapy options will provide valuable reference for our future research.

8. Tracking the patients’ quality of life and long-term adverse effects would offer insights into the tolerability of the regimen beyond the survival outcomes.

Thank you for your valuable suggestion. we acknowledge the importance of patients’ quality of life and long-term adverse effects to comprehensively assess the safety profile of the regimen. We will revise the discussion section to address this limitation and include it as a recommendation for future research

9. Mucositis was significantly more common in the CDFLME group; additional information on supportive care protocols and preventive measures for mucositis would be helpful.

Thank you for your suggestion. Mucositis is indeed a significant concern in the CDFLME group. We will provide additional details on the supportive care protocols and preventive measures for managing mucositis in our next update. Your feedback is valuable and will help us ensure comprehensive coverage of this important aspect.

10. The data availability statement restricts access due to institutional regulations. Providing a more accessible data-sharing plan, even with de-identified data, would enhance transparency and allow for independent verification of results.

Thank you for your feedback. We understand the importance of data accessibility for transparency and independent verification. While institutional regulations currently limit direct data sharing, we will explore options to provide de-identified data or alternative access methods within the allowed framework. Your suggestion is greatly appreciated and will guide us in improving future data-sharing plans.
