# Supplementary material for: Efficacy and safety of cisplatin + docetaxel + 5-FU + leucovorin + methotrexate and epirubicin combination chemotherapy for advanced esophageal cancer
Source: PLoS One. 2025 Jun 26;20(6):e0326056. doi: 10.1371/journal.pone.0326056 (PMC12200775; doi:10.1371/journal.pone.0326056)
Supplement: Attachment — Submitted filename: response_to_reviewer_auresp_2.docx [file pone.0326056.s003.docx]

Dear Editor and Reviewers

Thank you for spending your valuable time to review our paper. Fortunately, this paper could be submitted to PLOS one. We were grateful to Editors and Reviewers to review this paper and make some useful recommendation. That made this paper more valuable. It was our honor to have an opportunity to publish in PLOS one. We have considered the review’s comment and replied

them as below.

Review Comments to the Author

Reviewer #1: Dear authors,
I appreciate your addressing my previous comments. Nevertheless, the manuscript could still benefit from some revision. Below, I have provided my comments regarding its improvement.

1. Please define CDFLME when first used and remove the definition in the other part of the Introduction and Methods.

Thank you for your feedback. I have defined CDFLME at its first mention and removed the redundant definitions from the Introduction and Methods sections.

2. Please specify the exact sites you considered as “tumor progression” in the part you added to the methods section regarding tumor metastasis. If you have stated all of them, please substitute "including" for "such as."

Thank you for your suggestion. I have specified the exact sites considered as 'tumor progression' in the Methods section. I have replaced 'such as' with 'including' as recommended.

3. It is better to mention the date for the statement “J.-C. Lin designed a novel weekly CDFLME induction chemotherapy regimen” in the Introduction (e.g., In 2016, J.-C. Lin designed…)

Thank you for your suggestion. I have added the relevant date to the statement in the Introduction as recommended (e.g., 'In 2016, J.-C. Lin designed a novel weekly CDFLME induction chemotherapy regimen').

4. The definition of “partial response” in the RECIST guideline version 1.1 is as follows: At least a 30% decrease in the sum of diameters of target lesions, taking as reference the baseline sum diameters. Please revise it.

Thank you for your suggestion. I have revised the sentence for clarity as follows: ' partial response is defined as at least a 30% decrease in the sum of diameters of target lesions.'

5. Please provide a reference for the definitions of “treatment-related death” and “the disease control rate.”

Thank you for your suggestion. Since there is no consensus on a temporal definition of treatment-related death , we adopted the stricter criteria of Adelstein et al. (Adelstein DJ, Li Y, Adams GL, et al. An intergroup phase III comparison of standard radiation therapy and two schedules of concurrent chemoradiotherapy in patients with unresectable squamous cell head and neck cancer. J Clin Oncol 2003; 21: 92–98.) defining TRD as death occurring from CRT start until a month from the end of CRT.

Disease Control Rate (DCR): The proportion of patients who achieve a response to treatment, including complete response (CR), partial response (PR), and stable disease (SD), according to RECIST criteria

6. Please provide references for treatment schedules administered to both groups of patients in the Methods section.

Thank you for your suggestion. In the Methods section, the treatment schedule for the PF regimen has been referenced from [3], while the CDFLME regimen has been referenced from [17]

7. Please mention the explanation you stated in the “Author's Response To Reviewer Comments” section regarding informed consent in the Methods section as well (In this retrospective study, the requirement for informed consent was waived by the Institutional Review Board (IRB) of Changhua Christian Hospital (IRB number: 231101)).

Thank you for your suggestion. I have incorporated the explanation regarding informed consent into the Methods section as follows: 'In this retrospective study, the requirement for informed consent was waived by the Institutional Review Board (IRB) of Changhua Christian Hospital (IRB number: 231101)

8. Please provide the statement, “All monitoring visits were completed prior to the commencement of this retrospective study. The data used in this study were obtained from the medical records of Changhua Christian Hospital, ensuring accuracy and completeness” in the Methods section.

Thank you for your suggestion. I have added the following statement to the Methods section as recommended: 'All monitoring visits were completed prior to the commencement of this retrospective study. The data used in this study were obtained from the medical records of Changhua Christian Hospital, ensuring accuracy and completeness.

9. I couldn’t find “The results of the multivariate analysis confirmed that the CDFLME regimen was beneficial to overall survival” in the Discussion section.

Thank you for your feedback. I have added the following statement to the Discussion section: 'In the multivariate analysis, the results confirmed that the CDFLME regimen was beneficial for overall survival. This finding is consistent with our previous results for 1-year and 2-year OS. However, other factors, such as clinical stage, ECOG performance status, age, and tumor location, did not reach statistical significance, which may be due to the small sample size and selection bias

10. Please place both “Fig. 1” and “Fig. 2” where they are referred to in the text.

Thank you for your suggestion. I have adjusted the placement of 'Fig. 1' and 'Fig. 2' to align with their first mentions in the text.

11. In the Discussion section, please discuss all the variables you measured in your study and mentioned in the Results section.

Thank you for your suggestion. I have revised the Discussion section to ensure that all variables measured in the study and mentioned in the Results section are appropriately discussed.

12. Please mention the small sample size, the ratio of case and control groups, as well as not-matched groups for confounding factors, which result in a considerable selection bias, as limitations of your study

Thank you for your suggestion. I have added the following sentence to the Discussion section: 'The small sample size, the ratio of case and control groups, and the unmatched groups for confounding factors contribute to a considerable selection bias, which is a limitation of our study.

Reviewer #3: The author has responded thoroughly to reviewer comments, below is a general assessment and areas that still need improvement in the manuscript. After this reccomended for the publication.
General Comments:

1. While the authors acknowledge and explain the limitations of a small sample size and selection bias, the manuscript could further elaborate on how these issues might be mitigated in future studies.

Thank you for your suggestion. I have expanded the Discussion section to further elaborate on how the issues of small sample size and selection bias can be mitigated in future studies, including the potential use of randomized controlled designs and larger, more representative cohorts.
2. Despite assurances of language improvement, some sentences remain verbose or awkwardly phrased. Additional proofreading is necessary for clarity and grammatical correctness.

Thank you for your feedback. I will conduct additional proofreading to improve clarity and ensure grammatical correctness, refining any verbose or awkwardly phrased sentences
3. Although the authors agreed to include control numbers in the abstract, the revised text does not clearly specify this detail. Ensure this is updated.

Thank you for your suggestion. I have updated the abstract to clearly specify the control numbers as follows: ' Among them, 81 patients received fluorouracil + cisplatin regimen serving as the control group.' Please let me know if any further modifications are needed.
4. Moving figures to the results section as suggested is critical for better alignment with data presentation. Confirm that this change has been made appropriately.
Thank you for your suggestion. I have moved the figures to the Results section to better align with data presentation as recommended. Please let me know if any further adjustments are needed.
5. While the authors addressed inconsistencies like "CDFLEM" vs. "CDFLME" and "SCCHN" vs. "HNSCC," double-check that all terms are now consistent throughout the manuscript.

Thank you for your careful review. I have double-checked the manuscript to ensure that all terms, including 'CDFLME' and 'HNSCC,' are now consistently used throughout. Please let me know if any further modifications are needed.
6. The authors have clarified many points, but it is advisable to further detail why the chosen chemotherapy regimen is innovative compared to others, such as immune checkpoint inhibitors.
Thank you for your suggestion. The innovation of our chemotherapy regimen lies in its original use for head and neck cancer patients. In 2016, J.C. Lin demonstrated its efficacy in this population, which led to its small-scale application in esophageal cancer patients.
7. Adding a brief acknowledgment of the lack of quality-of-life metrics and why they were excluded (e.g., feasibility or retrospective nature) would strengthen the discussion.

Thank you for your suggestion. I have added a brief acknowledgment in the Discussion section as follows: Due to the fact that some patients continued their follow-up at branch hospitals of Changhua Christian Hospital, tracking quality-of-life metrics was challenging in terms of feasibility, leading to their exclusion.
8. The manuscript includes a restrictive data availability statement, which could be improved by providing clearer alternatives for data access.

Thank you for your feedback. In the future, we will communicate with the institution and explore clearer alternative solutions for data access to improve this aspect
