# Supplementary material for: Efficacy and safety of cisplatin + docetaxel + 5-FU + leucovorin + methotrexate and epirubicin combination chemotherapy for advanced esophageal cancer
Source: PLoS One. 2025 Jun 26;20(6):e0326056. doi: 10.1371/journal.pone.0326056 (PMC12200775; doi:10.1371/journal.pone.0326056)
Supplement: Attachment — Submitted filename: response_to_reviewer_auresp_3.docx [file pone.0326056.s004.docx]

Dear Editor and Reviewers

Thank you for spending your valuable time to review our paper. Fortunately, this paper could be submitted to PLOS one. We were grateful to Editors and Reviewers to review this paper and make some useful recommendation. That made this paper more valuable. It was our honor to have an opportunity to publish in PLOS one. We have considered the review’s comment and replied

them as below.

Reviewer #1: Dear authors,
I appreciate your addressing my previous comments. Nevertheless, the manuscript could still benefit from some revision. Below, I have provided my comments regarding its improvement.

1. CDFLME is still not defined correctly. Please notice that the Abstract and the body of the text are two different parts. Hence, CDFLME should be defined once in the Abstract and once in the body of the text, both when first used. Please consider this writing policy precisely.

Thank you for your feedback. I have revised the manuscript accordingly by defining CDFLME both in the Abstract and again at its first appearance in the main body of the text, as per the writing policy. Please let me know if any further adjustments are needed.

2. Regarding my previous comment, [“Please specify the exact sites you considered as “tumor progression” in the part you added to the methods section regarding tumor metastasis. If you have stated all of them, please substitute "including" for "such as."], still “including” is not substituted for “such as.” Please consider this carefully.

Thank you for your valuable comment. In accordance with your suggestion, we have replaced “such as” with “including” and clearly specified the sites considered indicative of tumor progression.

We have revised the sentence as follows: "When a tumor exhibits distant metastasis to sites including the lungs, liver, bones, distal lymph nodes, adrenal gland, or brain, it is considered disease progression."

3. Please add “taking as reference the baseline sum diameters” to the end of the statement “at least a 30% decrease in the sum of diameters of target lesions.”

Thank you for your suggestion. We have revised the sentence accordingly by adding “taking as reference the baseline sum diameters” to the end of the statement “at least a 30% decrease in the sum of diameters of target lesions,” as recommended.

4 .Unfortunately, I was unable to find a precise treatment schedule administered to the PF group in reference [3], as you mentioned. Furthermore, the treatment schedule described in reference differs from the one administered to the CDFLME group in your study in terms of the number of days.

Thank you for your comment. We acknowledge that the treatment schedule for the PF group was not clearly described in reference [3], as you pointed out. Additionally, we recognize that the schedule differed from that of the CDFLME group in terms of the number of treatment days.

To address this, we have replaced reference [3] with the NCCN Clinical Practice Guidelines in Oncology (NCCN Guidelines™) Esophageal Cancers and Esophagogastric Junction (excluding the proximal 5 cm of the stomach), Version 3.2023, which provides a more reliable and standardized reference.

Moreover, we have revised the description of the treatment schedule for the CDFLME group to clarify the differences in treatment days.

5.please remove the appended “in the Methods section” from the end of “All monitoring visits were completed prior to the commencement of this retrospective study. The data used in this study were obtained from the medical records of Changhua Christian Hospital, ensuring accuracy and completeness”.

Thank you for your suggestion. We have removed the appended phrase “in the Methods section” from the end of the sentence as requested.
