# Supplementary material for: Efficacy and safety of cisplatin + docetaxel + 5-FU + leucovorin + methotrexate and epirubicin combination chemotherapy for advanced esophageal cancer
Source: PLoS One. 2025 Jun 26;20(6):e0326056. doi: 10.1371/journal.pone.0326056 (PMC12200775; doi:10.1371/journal.pone.0326056)
Supplement: Attachment — Submitted filename: response_to_reviewer_auresp_4.docx [file pone.0326056.s005.docx]

Dear Editor and Reviewers

Thank you for spending your valuable time to review our paper. Fortunately, this paper could be submitted to PLOS one. We were grateful to Editors and Reviewers to review this paper and make some useful recommendation. That made this paper more valuable. It was our honor to have an opportunity to publish in PLOS one. We have considered the review’s comment and replied

them as below.

**Reviewer #1:** Dear authors,

I appreciate your addressing my previous comments. Nevertheless, the manuscript could still benefit from some revision. Below, I have provided my comments regarding its improvement.

1. Please clarify whether you administered the revised version of the treatment schedule for the CDFLME group or your previous schedule. Please note that it should be stated exactly as it was administered during the study. Furthermore, the new schedule is not consistent with Figure 1.

We appreciate the reviewer’s observation. We confirm that the treatment schedule administered to the CDFLME group was the original version, as described in the Methods section of the manuscript. We apologize for the confusion caused by the discrepancy between the revised schedule and Figure 1. To maintain consistency and accuracy, we have corrected Figure 1 to reflect the actual treatment protocol used during the study. All related descriptions have been revised accordingly in the revised manuscript.

2. Can you please provide the PDF of the NCCN Clinical Practice Guidelines in Oncology (NCCN GuidelinesTM) Esophageal Cancers and Esophagogastric Junction (Excluding the proximal 5cm of the stomach) Version 3.2023? I was unable to get access to it.

We sincerely thank the reviewer for pointing this out. Upon careful review, we acknowledge that we mistakenly cited the NCCN Clinical Practice Guidelines in Oncology (Version 3.2023) in our manuscript. In fact, the version we used and referenced throughout the study was **Version 2.2023**. We have corrected all citations accordingly in the revised manuscript to accurately reflect this. We apologize for the oversight and appreciate your attention to this detail.

The accurate citation is:

Ajani JA, D'Amico TA, Almhanna K, et al. NCCN Clinical Practice Guidelines in Oncology: Esophageal and Esophagogastric Junction Cancers, Version 2.2023. J Natl Compr Canc Netw. 2023 Apr;21(4):393–422. doi: 10.6004/jnccn.2023.0019.

We apologize for the oversight and appreciate your attention to this detail.
